# Highly Porous and Ultra-Lightweight Aero-Ga_2_O_3_: Enhancement of Photocatalytic Activity by Noble Metals

**DOI:** 10.3390/ma14081985

**Published:** 2021-04-15

**Authors:** Irina Plesco, Vladimir Ciobanu, Tudor Braniste, Veaceslav Ursaki, Florian Rasch, Andrei Sarua, Simion Raevschi, Rainer Adelung, Joydeep Dutta, Ion Tiginyanu

**Affiliations:** 1 National Center for Materials Study and Testing, Technical University of Moldova, Stefan cel Mare Av. 168, MD-2004 Chisinau, Moldova; vladimir.ciobanu@cnstm.utm.md (V.C.); tudor.braniste@cnstm.utm.md (T.B.); vvursaki@gmail.com (V.U.); 2 Functional Nanomaterials, Institute for Materials Science, Kiel University, Kaiser Str. 2, 24143 Kiel, Germany; flce@tf.uni-kiel.de (F.R.); ra@tf.uni-kiel.de (R.A.); 3 H. H. Wills Physics Laboratory, School of Physics, University of Bristol, Tyndall Avenue, Bristol BS8 1TL, UK; a.sarua@bristol.ac.uk; 4 Department of Physics and Engineering, State University of Moldova, Alexei Mateevici Str. 60, MD-2009 Chisinau, Moldova; raevskis@mail.ru; 5 Functional Materials Group, Applied Physics Department, School of Engineering Sciences, KTH Royal Institute of Technology, Hannes Alfvéns väg 12, 11419 Stockholm, Sweden; joydeep@kth.se; 6 Academy of Sciences of Moldova, Stefan cel Mare Av. 1, MD-2001 Chisinau, Moldova

**Keywords:** aeromaterial, Ga_2_O_3_, photocatalysis, metal-semiconductor photocatalyst, methylene blue degradation

## Abstract

A new type of photocatalyst is proposed on the basis of aero-β-Ga_2_O_3_, which is a material constructed from a network of interconnected tetrapods with arms in the form of microtubes with nanometric walls. The aero-Ga_2_O_3_ material is obtained by annealing of aero-GaN fabricated by epitaxial growth on ZnO microtetrapods. The hybrid structures composed of aero-Ga_2_O_3_ functionalized with Au or Pt nanodots were tested for the photocatalytic degradation of methylene blue dye under UV or visible light illumination. The functionalization of aero-Ga_2_O_3_ with noble metals results in the enhancement of the photocatalytic performances of bare material, reaching the performances inherent to ZnO while gaining the advantage of the increased chemical stability. The mechanisms of enhancement of the photocatalytic properties by activating aero-Ga_2_O_3_ with noble metals are discussed to elucidate their potential for environmental applications.

## 1. Introduction

Five different polymorphs have been reported for gallium oxide (Ga_2_O_3_), namely, the monoclinic (β), rhombohedral (α), defective spinel (γ), cubic (σ), and orthorhombic (ε) structures [1,2]. β-polymorph Ga_2_O_3_ has attracted most of the attention due to its superior chemical and thermal stability, wide bandgap, high stability to breakdown voltage, and high Baliga’s figure of merit (BFOM). It has been widely studied and utilized for various applications including in power electronics, solar blind UV photodetectors, solar cells, and as gas-sensing materials [3,4,5]. Photocatalysis is another emerging application of the β-Ga_2_O_3_ polymorph. Particularly, the photocatalytic activity of the Ga_2_O_3_ polymorphs was found to be strongly influenced by its crystal structure in the following order: β-Ga_2_O_3_ > α-Ga_2_O_3_ > γ-Ga_2_O_3_ [6].

Ga_2_O_3_-based pure phases and composites have been examined for energy and environmental applications, including the decomposition of volatile aromatic pollutants in air [6]; water purification [7,8,9,10,11]; solar water splitting [12,13,14,15]; photocatalytic carbon dioxide (CO_2_) reduction with water to produce carbon monoxide (CO), hydrogen (H_2_), and oxygen (O_2_) [15,16,17,18,19,20,21]; photocatalytic reduction of CO_2_ to produce methane (CH_4_) [22]; as well as solar-driven photoreduction of nitrogen (N_2_) in a clean route to produce ammonia (NH_3_) [23].

Generally, three main factors determine the solar-to-chemical energy conversion efficiencies of photocatalytic processes: (i) light absorption to produce photogenerated charge carriers; (ii) transfer and separation of charge carriers; (iii) surface reactions to convert reactants into products through the consumption of charge carriers [24]. Therefore, the use of a single semiconductor material is limited by these key factors, since their synergistic combination is rarely found in the same material.

Different approaches have been proposed for enhancing the photocatalytic performance of catalysts, such as making use of co-catalysts, the development of semiconductor-based hybrid photocatalysts, crystal phase engineering, and the rational design of phase junctions [24], e.g., via implementing heterojunctions [25,26,27,28]. Furthermore, coupling photocatalysts with conductive materials and utilizing the surface plasmon resonance (SPR) to produce plasmonic photocatalysis [26,27,28,29,30] show promising outcomes. By implementing these approaches, the following issues were addressed: (i) the light absorption region was extended by combining various photosensitizers with semiconductors, particularly by deposition of nanoparticles (NPs) of noble metals such as gold (Au), silver (Ag), and platinum (Pt) to enhance visible light absorption due to SPR; (ii) suppression of electron–hole recombination through efficient charge separation and confinement of the photogenerated electrons and holes in different components of semiconductor-based heterostructures or by using conductive materials, particularly noble metal NPs or carbon materials as electron acceptors and traps to enhance the carrier separation in photocatalysts and to avoid the recombination of charges; (iii) surface reactions were enhanced by integrating co-catalysts with semiconductors.

Nevertheless, the photocatalytic systems developed to date are still far from being applicable due to low efficiency and poor durability [25]. Particularly, the chemical stability of photocatalysts, including that of the most widely explored metal oxides as titanium dioxide (TiO_2_) and zinc oxide (ZnO) materials, presents a major challenge for practical applications [26,31].

In this research, we focused on the development of an efficient photocatalyst, which will not decompose in the process, bringing additional water pollution with metal ions or new compounds. The nanostructured titania and zinc oxide are the undebatable leaders among the semiconductor photoactivated catalysts. At the same time, sewage and ground waters suffer much from the deliberate usage of soaps, medicines, and cosmetics containing TiO_2_ and ZnO. According to the WHO reports, content of Zn in tap water can cover 10% of the daily amount of this mineral in human body, but taking into account its high accessibility from meat, fish, and cereals, this limit may be exceeded. Ingestion of excessive amounts of Zn causes fever, nausea, vomiting, stomach cramps, and diarrhea at humans, decreases the antibiotics effectiveness, etc. [32]. It was reported that intake of Zn overdoses for a long period of time increases the risks to develop prostate cancer [33]. Thus, the major concern of modern research is the development of sustainable technologies that are efficient and cost-effective but also with low level of toxicity. 

In this work, we report on the design of an ultra-lightweight, highly porous, and stable aero-Ga_2_O_3_ material and demonstrate the photocatalytic efficiency for potential applications in photocatalytic water purification.

## 2. Materials and Methods

The aero-Ga_2_O_3_ material belongs to a class of highly porous and ultra-lightweight “aero-materials” which descend from 3D semiconductor network of interpenetrating ZnO microtetrapods. The sacrificial network of ZnO microtetrapods was prepared by a simple flame transport approach, which is described elsewhere [34]. So far, new aero-materials such as aerographite [35], aero-GaN [36,37,38], aero-ZnS [39], aero-BN [40], and aero-Si [41] have been realized by templating the ZnO network. For example, the aerographite is produced via the transformation of the sacrificial ZnO microtetrapod network into graphitic microtubes in a one-step chemical vapor deposition (CVD) process with toluene as the carbon source [35].

### 2.1. Materials Synthesis

**Figure 1:**
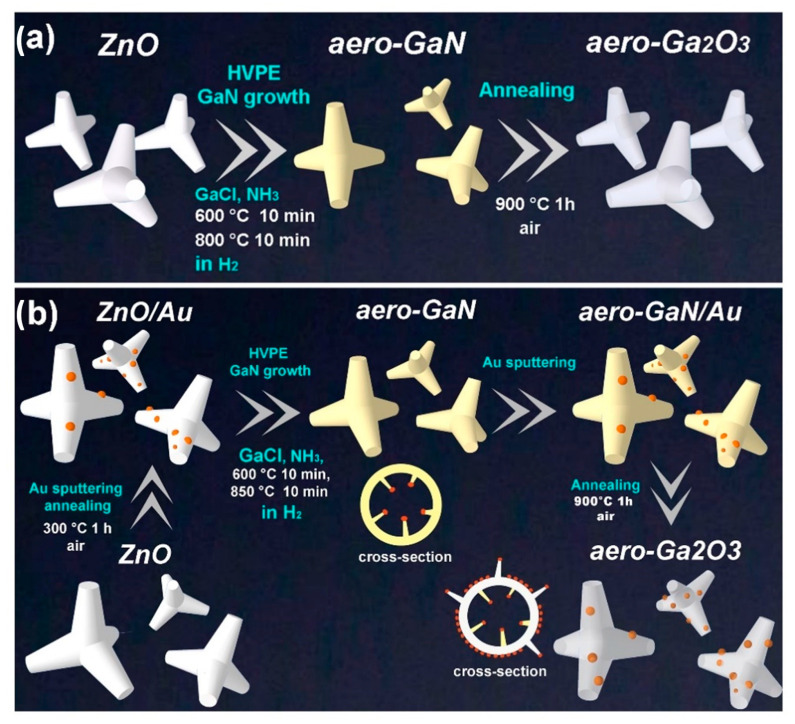
(**a**) Schematic representation of the technological routes for the preparation of aerogallox, (**b**) and aero-Ga_2_O_3_-Au hybrid photocatalyst.

The new aero-Ga_2_O_3_ is produced by a two-step process schematically represented in Figure 1a. Aero-GaN is first obtained by transforming the ZnO microtetrapods into GaN microtubes in a hydride vapor phase epitaxy (HVPE) process using hydrochloride (HCl), metallic gallium (Ga), and ammonia precursors as described in previous reports [36,37,38]. Gallium chloride (GaCl) is formed in the source zone, where gaseous HCl interacts with liquid Ga in the first stage of this process, while GaN is formed in the reaction zone via a chemical reaction between the gaseous molecules of GaCl and NH_3_. Simultaneously, the ZnO sacrificial template is decomposed due to the corrosive atmosphere and high temperatures. Secondly, the aero-GaN is subjected to annealing at 900 °C for 1 h under normal atmospheric conditions. As a result, aero-GaN is transformed into aero-Ga_2_O_3_ (also known as “Aerogallox”) [42]. 

Here, samples were prepared via the second hybrid approach, which is similar to that applied for the fabrication of the phase pure aerogallox, but it is complemented by the deposition of Au or Pt coatings in two technological steps. The first coating is deposited on the ZnO template before the HVPE process is performed for the production of aero-GaN. Following this, the second coating is deposited on the aero-GaN architecture before the annealing is performed for the transformation of aero-GaN into aero-Ga_2_O_3_. Thin gold or platinum films were deposited in a Cressington 108 Sputter Coater machine as described in a previous paper [43]. The thermal treatment leads to the structuring of the initially continuous metal film and to the formation of hybrid photocatalysts.

### 2.2. Materials Characterization

The microstructure morphology of aero-Ga_2_O_3_ microtetrapods was studied by scanning electron microscopy (SEM) Zeiss Gemini Ultra55 Plus (Carl Zeiss AG, Oberkochen, Germany) working at 7 kV. Raman spectra were recorded using a Renishaw InVia Raman system (Renishaw plc, Wotton-under-Edge, UK) in backscattering geometry at room temperature. The samples were illuminated using a 532 nm line of a CW DPSS laser with a power density of 11.3 mW/µm^2^. A 50× microscope objective lens with NA = 0.75 was used to focus the light on the sample surface. Raman spectra were collected from a single gallium oxide tube where possible with light normal to the side wall. The scattered light was detected by a cooled charge-coupled device detector. 

A JEOL 6330F (JEOL Ltd., Tokyo, Japan) field emission scanning electron microscope (FE-SEM) equipped with a Gatan MonoCL cathodoluminescence (CL) microanalysis system was used for CL characterization. The CL spectra have been taken with an accelerating voltage of 10 keV and current of 10 nA in the spectral range of 250–600 nm, using a grating spectrometer and a photomultiplier tube (PMT) detector.

### 2.3. Photocatalytic Degradation of MB Solution

Methylene blue (MB) dye (Merck KGaA, Darmstadt, Germany) was chosen for investigating the photocatalytic properties of the developed catalyst, since it is a common organic pollutant recommended by the International Standards Organization ISO 10678: 2010. A 10 μM MB solution in deionized (DI) water was prepared as the organic contaminant. Consequently, 50 mL of MB solution was transferred into a glass beaker with 20 mg of catalyst and mixed at 600 rpm by a magnetic stirrer. The same concentration of 0.4 mg/mL of the active material in solution was used for all the tested photocatalysts. The solution with aero-material was placed under a 100 W Blak Ray Hg lamp (Analytik Jena GmbH, Jena, Germany) with the main intensity peak at 365 nm, at 14.5 cm distance from the solution surface to study the photocatalytic properties under ultraviolet (UV) illumination, or under a 150 W Halogen lamp irradiation ensuring an optical power density of 100 mW/cm^2^ to estimate the photodegradation with visible light. To monitor the degradation of MB, the samples were transferred into cuvettes for UV/Vis spectroscopy, and the absorption spectra were recorded with a Perkin Elmer Lambda 750 UV/Vis spectrometer (PerkinElmer Life and Analytical Sciences, Shelton, CT, USA). By monitoring the absorption intensity decay as a function of time, we calculated the concentration of remaining MB in the solution. The MB absorption peak was observed at 665 nm and the current concentration of MB was calculated using Beer–Lambert law:

(1) cMB=Aεl

where $c_{MB}$ is the solution concentration, *A* is the measured absorption value, *ε* is the absorptivity of the solution at certain wavelength (λ), and *l* is the optical pathway during the measurement expressed in centimeters. The absorptivity of dye ε has been extracted from the blank test data.

The MB degradation experiments were performed at low pollutant concentration; thus, the kinetics study was performed according to the first-order Langmuir–Hinshelwood model that relates the rate of photochemical reactions, which are proportional to the surface coverage of the photocatalyst:

(2) −ln(CMBC0)=K

where *K* is the adsorption coefficient of the reactant on the surface of the catalyst, $c_{MB}$ is the solution concentration, and $c_{0}$ is the initial pollutant concentration.

## 3. Results and Discussions

### 3.1. Morphology of the Aero-Ga_2_O_3_

**Figure 2:**
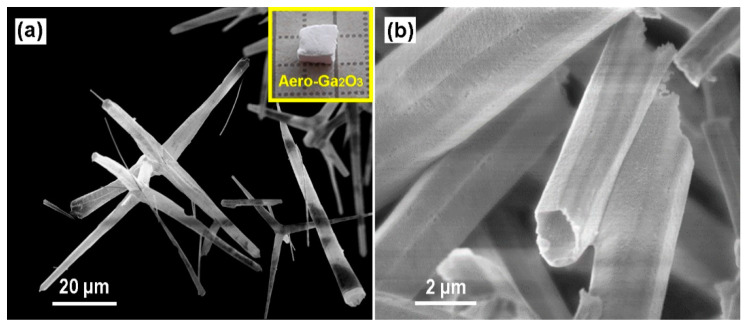
(**a**) SEM micrograph of aero-Ga_2_O_3_ microtetrapods, (**b**) Magnified micrograph revealing the surface features of the microtetrapod surface. The inset in (**a**) shows a photograph of Aero-Ga_2_O_3_.

An SEM micrograph of the aero-Ga_2_O_3_ material used for photocatalytic degradation tests is presented in Figure 2a. The aero-Ga_2_O_3_ microstructure displays a network of interconnected microtetrapods. The Ga_2_O_3_ tetrapods preserve the initial shape of the ZnO template; however, they are converted into a hollow geometry. Concerning the crystallographic structure of the obtained Ga_2_O_3_ material, it was shown in previous work to belong to the β-Ga_2_O_3_ polytype with the *C2*/*m* (*C^3^_2h_*) space group [42]. This assignment was confirmed by the Raman scattering analysis discussed below.

**Figure 3:**
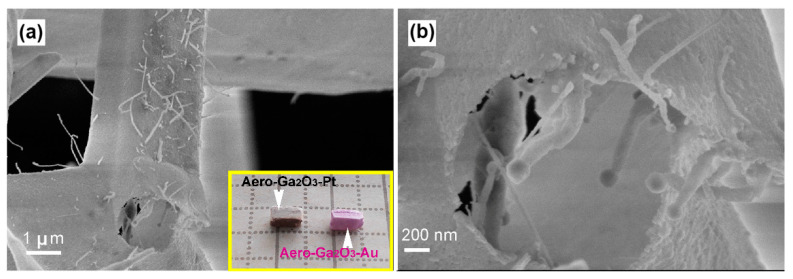
(**a**) SEM image of an aero-Ga_2_O_3_-Au microtetrapod, (**b**) Magnified image of the microtube opening in section (**a**). The inset in (**a**) shows photographs of Aero-Ga_2_O_3_ samples functionalized with noble metals.

The morphology of the aero-Ga_2_O_3_-Au hybrid photocatalyst (Figure 3) is similar to that of the pure Ga_2_O_3_. However, an array of Ga_2_O_3_ nanowires (NWs) terminated by Au nanoparticles grows inside the Ga_2_O_3_ microtubes during the HVPE on sacrificial ZnO microtetrapods, as illustrated in Figure 3b. The growth of these nanowires was elucidated in detail in a previous paper [43]. It was shown that the confined reaction conditions during the HVPE process and hydrothermal dissolution of ZnO lead to the metal-catalytic vapor–liquid–solid (VLS) growth of NWs. Some nanowires with golden nanoparticles on top are also observed on the outer surface of aero-Ga_2_O_3_-Au, which were formed during the last step of the oxidation of GaN microtubes after being covered with an ultrathin layer of Au nanostructures. However, the aero-Ga_2_O_3_-Au and aero-Ga_2_O_3_-Pt hybrid photocatalysts are basically composed of Ga_2_O_3_ microtubes with noble metal nanocoatings. 

### 3.2 Optical Properties

**Figure 4:**
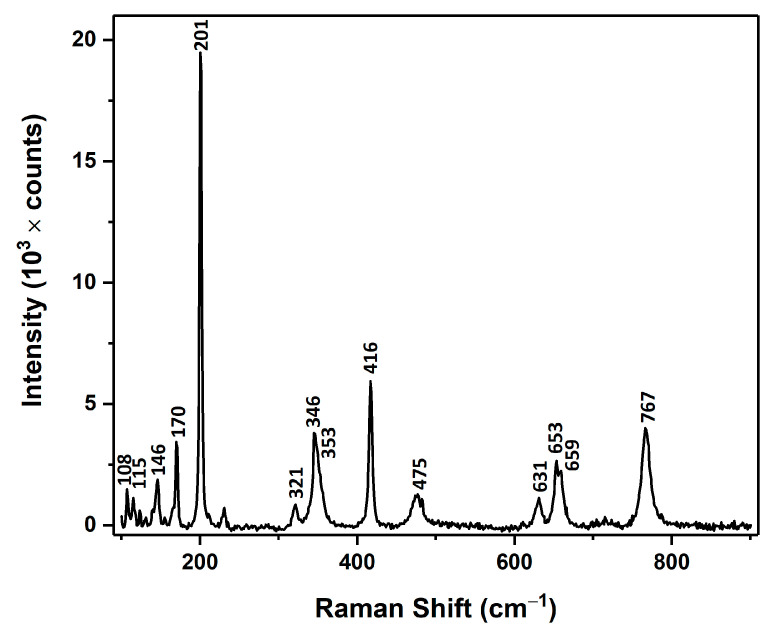
Raman spectrum of aero-Ga_2_O_3_ measured at room temperature.

As mentioned below, the Raman spectrum of the aero-Ga_2_O_3_ (Figure 4) corroborates well with its attribution to the β-Ga_2_O_3_ monoclinic polytype. The primitive unit cell of β-Ga_2_O_3_ consists of 10 atoms at the Γ-point with irreducible representation Γ_opt_ = 10Ag + 5Bg + 4Au + 8Bu predicts a set of 27 optical modes of which 15 g modes are Raman-active and 12 u modes are IR-active only [44]. 

All the Raman active modes are observed in the measured Raman spectrum, which are summarized in Table 1 along with the classification given in Ref. [44] and refs therein.

The frequencies of the A_g_^(7)^ and B_g_^(4)^ modes coincide. A series of weaker peaks are also observed at 123 cm^−1^, 131 cm^−1^, 140 cm^−1^, 155 cm^−1^, 166 cm^−1^, 211 cm^−1^, 231 cm^−1^, and 482 cm^−1^ in the spectrum (Figure 4), which can be attributed to either activation of Raman inactive modes due to breaking of local symmetry, to some local vibrational modes, or to second-order Raman modes. The Raman spectra were not affected by metal deposition, and no vibration modes related to metal inclusions were observed in the spectrum.

**Figure 5:**
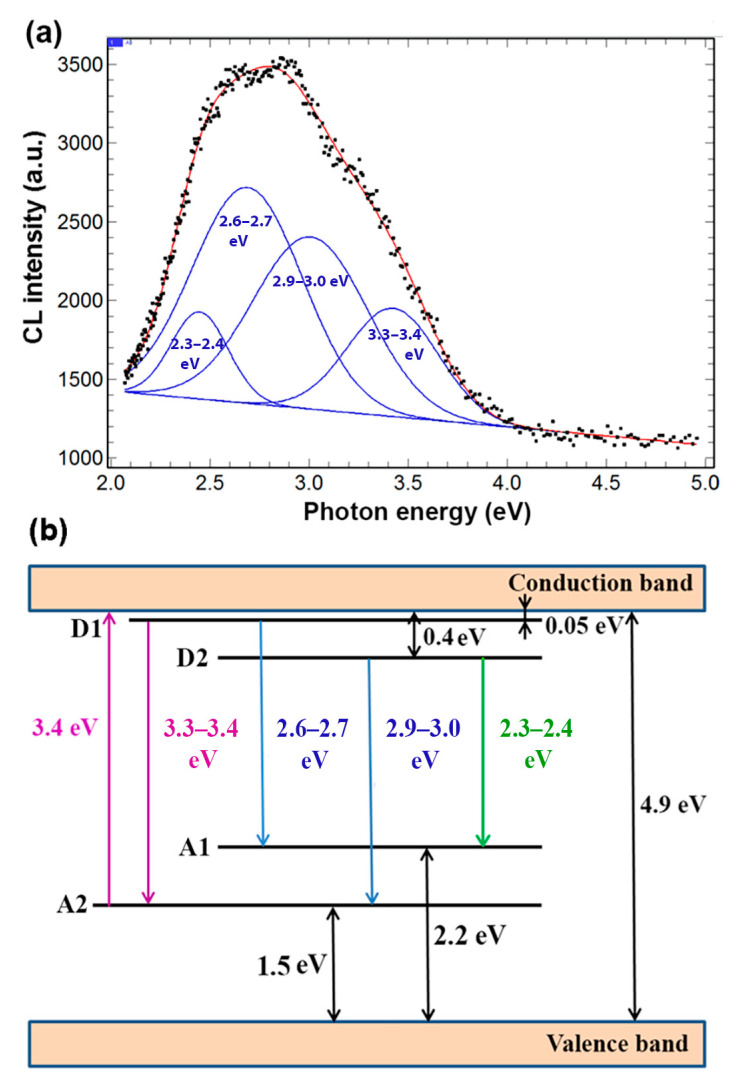
(**a**) Measured and deconvoluted cathodoluminescence (CL) spectrum of aero-Ga_2_O_3_ and (**b**) schematic diagram of energy bands and electron transitions in aero-Ga_2_O_3_.

The presence of donor and acceptor centers in the prepared aero-Ga_2_O_3_, their energy levels, and the corresponding electron transitions can be deduced from the cathodoluminescence spectrum (Figure 5a). The emission spectrum is deconvoluted into four Gaussian CL bands with maxima around (3.3–3.4) eV, (2.9–3.0) eV, (2.6–2.7) eV, and (2.3–2.4) eV. The maxima of CL bands and the position of respective energy levels were determined with an uncertainty of around 5%. One should also take into consideration that the position of the luminescence band related to distant donor–acceptor pair recombination depends upon the excitation power density used in the experiment. The luminescence spectra were not affected by metal deposition in the materials reported in this paper. 

The scheme of energy levels and electron transitions plotted according to the observed CL bands is presented in Figure 5b. This scheme contains two donor and two acceptor levels, which is in accordance with the model proposed by Mi et al. [45]. 

According to this model, the two blue emission bands, at (2.6–2.7) eV and (2.9–3.0) eV in our case, arise from electron transitions from the D1 to the A1 level and from the D2 to the A2 level, respectively. The UV emission band at (3.3–3.4) eV was attributed to the recombination of an electron on the D1 donor level with a hole on the A2 acceptor level, while the green band at (2.3–2.4) eV was associated with electron transition from the D2 to the A1 level. It was suggested that the donor levels can be formed by oxygen vacancies (V_O_^X^) and Ga^2+^ interstitials, while the acceptor levels can be attributed to gallium vacancy (V_Ga_^X^) and gallium–oxygen vacancy pairs [(V_Ga_,V_O_)^X^] [45,46,47]. The PL bands at 2.4, 2.7, and 3.0 eV have been supposed to arise from donor–acceptor pair recombination involving the same donor, while acceptors are associated with interstitial oxygen (O_i_^0^), gallium vacancy (V_Ga_^2−^), and gallium–oxygen vacancy pairs [(V_Ga_,V_O_)^1−^], respectively [48]. The acceptors involved in the donor–acceptor pair recombination generating the green emission band at 2.3 eV were also associated with either interstitial oxygen (O_i_^0^), octahedral gallium vacancy (V_Ga_^2−^), or tetrahedral gallium vacancy (V_Ga_^1−^) [49].

The prepared aero-Ga_2_O_3_ material as well as the aero-Ga_2_O_3_-metal hybrid structures were subjected to photocatalytic tests under UV and visible light illumination in order to degrade the MB solution. The effect of a wide range of photocatalysts on the degradation and discoloring of MB was extensively investigated previously, including those based on β-Ga_2_O_3_ [50,51]. Upon excitation by the UV light with a wavelength of 365 nm, an electron from the acceptor level A2 is excited into the conduction band, as shown in Figure 5b. As a result of this transition, an electron from the valence band non-radiatively recombines with the hole formed on the acceptor level, thus leaving a free hole in the valence band. The holes in the valence band are able to oxidize (OH^−^) in reaction with water to produce reactive hydroxyl radicals (•OH). On the other hand, the excited electrons in the conduction band are able to produce superoxide anion radicals (O_2_^•−^) upon reacting with O_2_. Both (•OH) and (O_2_^•−^) are free radicals and being strong oxidants are able to mineralize organic and inorganic carbon compounds producing carbon dioxide, water, and other smaller organic molecules [8,10,27,28,51,52,53].

### 3.3 Photocatalytic Performance

**Figure 6:**
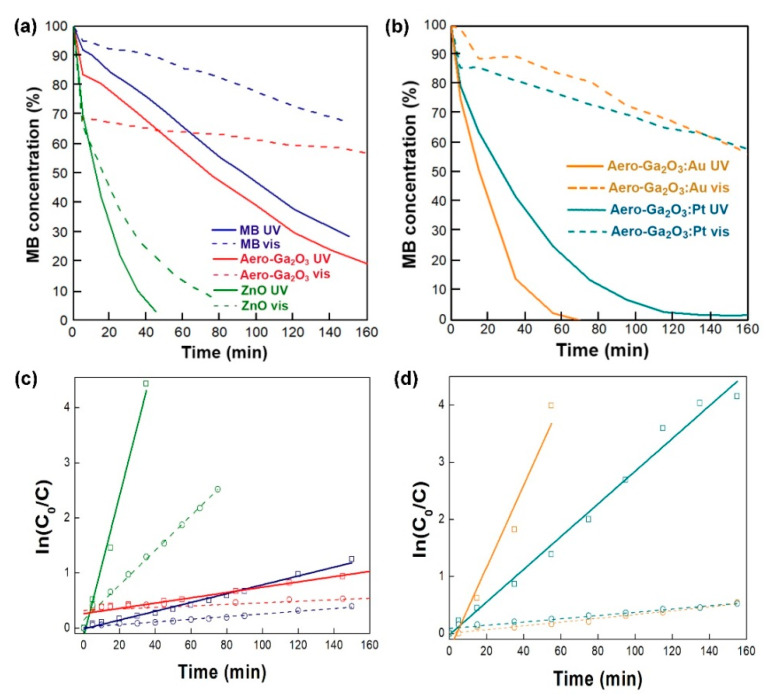
Comparison of photocatalytic activities under UV and visible light illumination of the prepared aero-Ga_2_O_3_ material and the initial ZnO template (**a**), and of aero-Ga_2_O_3_-Au and aero-Ga_2_O_3_-Pt photocatalysts (**b**), the kinetics of the photodegradation is presented in (**c**,**d**) plots corresponding to (**a**,**b**) methylene blue (MB) concentration evaluation. The concentration of the catalyst was maintained at the level of 0.4 mg/mL in all cases.

The evolution of the pollutant concentration during the experiments and photocatalytic rate constant were calculated according to Equations (1) and (2), respectively, and the resulting plots are presented in Figure 6. The photocatalytic activity of the aero-Ga_2_O_3_ without metal activation is compared in Figure 6a with that of the initial ZnO microtetrapod template. The high activity of ZnO under visible light illumination led to 90% degradation of MB dye within 60 min, while under UV excitation, 90% of the dye is degraded within 35 min. On the other hand, the aero-Ga_2_O_3_ performs worse, with only 35% degradation of dye observed after 45 min both under visible and UV light illumination, while only 43% was degraded after 60 min under UV excitation.

The performance of the aero-Ga_2_O_3_ was significantly improved by noble metal activation, as shown in Figure 6b, so that the aero-Ga_2_O_3_-Au hybrid structure degraded about 85% of the dye within 35 min under UV excitation, while the aero-Ga_2_O_3_-Pt composite degraded 60% after the similar exposure time under UV illumination. After a 60 min exposure, the dye was almost completely degraded by the aero-Ga_2_O_3_-Au hybrid structure, while 80% of the dye was degraded by the aero-Ga_2_O_3_-Pt composite. The photocatalysts did not promote any noticeable degradation under visible light illumination compared to the natural dye degradation.

Adsorption rates of MB on aero-Ga_2_O_3_ bare and functionalized with Au and Pt were analyzed using the pseudo 1st order kinetic model, according to Equation (2). Plots of ln(C0CMB) versus the time of reaction are presented in Figure 6c,d. The rate constants obtained from the slopes of the lines in Figure 6 in case of no catalyst, aero-Ga_2_O_3_, aero-Ga_2_O_3_–Pt, aero-Ga_2_O_3_–Au, ZnO tetrapodes under UV and visible light illumination are presented in Table 2. 

As mentioned above, two mechanisms are expected to contribute to the enhancement of photocatalytic activity of semiconductors by noble metal functionalization: namely, the extension of the light absorption region by surface plasmon effects and the suppression of charge recombination due to carrier separation at the metal-semiconductor Schottky contact. The surface plasmon resonance frequencies of gold nanoparticles and films embedded in various semiconductor matrices were found to be in the spectral range of 500–700 nm [54,55,56,57,58,59]. The resonance frequencies of platinum are also in the visible light spectrum [60,61]. However, according to very low catalytic activity under visible light illumination of aero-Ga_2_O_3_ functionalized with Au or Pt, as deduced from Figure 6b, one can conclude that light absorption is not extended to the visible light spectrum, indicating that the plasmonic effects of Au or Pt coatings are negligible in the prepared aero-Ga_2_O_3_ catalyst. On the contrary, the improvement of the catalytic performance upon Pt deposition, and especially by Au functionalization, may come from effective carrier separation at the Schottky contact formed at the semiconductor metal interface, especially when the noble metal is in the form of dots [62]. 

According to previously published data, the work function of Au was estimated to be of 5.2–5.3 eV [63,64], while the reported value for Pt was in the range of 5.6–5.9 eV [3,64]. A value of 4.0 eV was reported for the electron affinity of Ga_2_O_3_ leading to the formation of a Schottky barrier height of 1.2 eV at the Au/Ga_2_O_3_ interface according to the Schottky–Mott rule [3,5,63,65]:

(3) $$\Phi_{B}=\Phi_{Au}-\chi$$

where *Φ_B_* is the barrier height, *Φ_Au_* is the Au work function, and *χ* is the electron affinity of β-Ga_2_O_3_.

The Schottky barrier height for the Pt/Ga_2_O_3_ interface should be a little higher. However, the real Schottky barrier height is also affected by the Fermi-level pinning at the metal-semiconductor interface and by the chemical disorder, so that the measured value of the barrier height usually differs from the calculated one. For instance, the measured value of Schottky barrier height was in the range of 1.0–1.7 eV for Au [3,63,64,65], and 1.0–1.6 eV for Pt [3,64,65,66]. Thus, considering the obtained photodegradation results with aero-Ga_2_O_3_-Au and aero-Ga_2_O_3_-Pt catalysts, we conclude that the aero-Ga_2_O_3_ composite with Au provides higher catalytic activity, which is most likely due to the higher Schottky barrier and carrier blocking for Au, as compared to that with Pt. The better quality of the contact ensures more efficient charge separation at the interface and suppression of free carrier recombination, which in its turn results in a higher photocatalytic activity of the Ga_2_O_3_-Au photocatalysts. The performance of this photocatalyst is similar to that obtained with the initial ZnO microstructured template, but the aero-Ga_2_O_3_ material is much more stable in contact with various chemicals compared to ZnO [67,68].

The catalysts presented in this study have been tested for several runs. After the first test, the catalysts were centrifuged, washed in DI, centrifuged again, and dried at 100 °C in air, and tests were repeated in a new run maintaining the catalyst concentration in solvent of 0.4 mg/mL. It was observed that material keeps its performance on the fair level after being reused.

## 4. Conclusions

The results of this study demonstrate the potential of the newly developed aero-Ga_2_O_3_-Au hybrid structure for environmental applications. Good crystallinity of the β-Ga_2_O_3_ phase of microtubes constituting the aero-Ga_2_O_3_ architecture was demonstrated by Raman scattering spectroscopy. The scheme of energy bands and electron transitions in aero-Ga_2_O_3_ deduced from CL spectra suggests the existence of effective channels for UV excitation with the 365 nm line of the aero-Ga_2_O_3_ matrix with the subsequent formation of (•OH) and (O_2_^•−^) free radicals in water, which are strong oxidants that are able to oxidize the MB dye. The photocatalytic activity of the pure aero-Ga_2_O_3_ material is behind the performances of the initial ZnO microtetrapods-based template, while the functionalization of the aero-Ga_2_O_3_ with noble metals results in spectacular enhancement of the photocatalytic performances of this new material. The performed analysis suggests that the main contribution to this enhancement comes from the formation of Schottky barriers at the Au or Pt /aero-Ga_2_O_3_ interface leading to effective separation of the excited free carriers and suppression of their recombination. Although the performance of the developed photocatalyst is at the level inherent to the initial ZnO template, the aero-Ga_2_O_3_ functionalized with noble metals represents a promising composite material exhibiting high chemical stability and possessing a unique spatial architecture.

## Figures and Tables

**Table 1:** Spectral position of the Raman peaks of β-Ga_2_O_3_, given in cm^−1^.

Phonon Mode	This Work	Ref. [44]
Ag(1)	108	111.0
Bg(1)	115	114.8
Bg(2)	146	144.8
Ag(2)	170	169.9
Ag(3)	201	200.2
Ag(4)	321	320.0
Ag(5)	346	346.6
Bg(3)	353	353.2
Ag(6)	416	416.2
Ag(7)	475	474.9
Bg(4)	475	474.9
Ag(8)	631	630.0
Bg(5)	653	652.3
Ag(9)	659	658.3
Ag(10)	767	766.7

**Table 2:** Kinetic data of MB photodegradation on UV/vis illumination in the presence of catalysts.

Catalyst	*k* (Rate Constant)	R^2^ (Linear Coefficient Regression)
MB (UV)	0.0080	0.9882
Aero-Ga_2_O_3_ (UV)	0.0048	0.9418
Aero-Ga_2_O_3_-Pt (UV)	0.0286	0.9877
Aero-Ga_2_O_3_-Au (UV)	0.7192	0.9588
ZnO (UV)	0.1270	0.9888
MB (vis)	0.0024	0.9803
Aero-Ga_2_O_3_ (vis)	0.0014	0.5090
Aero-Ga_2_O_3_-Pt (vis)	0.0028	0.9502
Aero-Ga_2_O_3_-Au (vis)	0.0033	0.9760
ZnO (vis)	0.0310	0.9930

## Data Availability

The data presented in this study are available on request from the corresponding authors.
